# Love (and) ageing well: A qualitative study of sexual health in the context of ageing well among women aged 50 and over

**DOI:** 10.1177/17455057241247747

**Published:** 2024-04-29

**Authors:** Sophie Patterson, Kate Jehan

**Affiliations:** 1 Faculty of Health and Medicine, Lancaster University, Lancaster, UK; 2 Faculty of Health Sciences, Simon Fraser University, Burnaby, BC, Canada; 3 Department of Public Health and Policy, Liverpool University, Liverpool, UK

**Keywords:** ageing well, public health, qualitative research, sexual health, women

## Abstract

**Background:**

The United Nations has declared 2021–2030 the ‘Decade of Healthy Ageing’ and identified the need to strengthen the evidence base on interpretations and determinants of healthy ageing to inform policy.

**Objectives:**

This study sought to interrogate a ‘policy blind spot’ and examine interpretations and experiences of sexuality and sexual health within the context of ageing well among women aged 50+.

**Design:**

The qualitative study design was underpinned by an interpretivist epistemology. Research was guided by principles of feminist scholarship and located in an affirmative ageing framework.

**Methods:**

Semi-structured individual interviews were conducted between April–June 2019 with 21 English-speaking women aged 52–76. Women were recruited through community organizations in North West England. Transcripts were analysed using a framework approach to thematic analysis, applying an inductive approach to theme generation.

**Results:**

Narratives encompassed six broad themes: reflections on ‘ageing well’; age alone does not define sexuality and sexual health; interpretations of sexual health and sexuality; vulnerability and resistance in later-life sexual health; narratives of (in)visibility; and reimagining services to promote sexual health in later life. There was a dominant belief that sexual health represents a component of ageing well, despite a broad spectrum of sexual expression and health challenges. Sexual expression was diversely shaped by conflicting societal expectations within an evolving digitized environment. In clinical settings, however, sexual health discussions were often muted or framed from a disease-focussed lens. Women expressed a preference for holistic, person-centred sexual health provision from an orientation of wellness to support varied sexual expression, sensitive to wider health, life and relationship realities.

**Conclusion:**

This work strengthens calls to disentangle sexual health from disease-centred narratives and legitimize sexual health as part of the healthy ageing agenda.

## Introduction

The United Nations (UN) has declared 2021–2030 the ‘Decade of Healthy Ageing’ with a goal of improving the health and lives of the older population.^1,2^ In 2020, 9% of global citizens were aged 65+ and predicted to escalate to 16% by 2050.^1^ This shifting demographic picture has significant implications for health services, the economy and society more broadly, realigning global priorities for public health and policy making.

In the 2015 World Report on Ageing and Health, the World Health Organization (WHO) outlined a public health framework to inform the response to our ageing global population.^3^ This report decoupled healthy ageing from the presence or absence of disease, defining it as the ‘ongoing process of developing and maintaining the functional ability that enables wellbeing in older age’.^3^ Functional ability was broadly interpreted by the WHO as ‘health-related attributes that enable older people to be and to do what they have reason to value’, shaped by physical and mental capacity, but also the broader social and physical environment. If well-being is the desired outcome of healthy ageing, clarifying interpretations and determinants of well-being in later life becomes a critical step towards addressing priority areas of action within the UN’s Decade of Healthy Ageing.^2,4^ However, the relevance of sexual health (SH) in later-life well-being is often overlooked.^5,6^

The WHO defines SH as

. . . a state of physical, emotional, mental and social well-being in relation to sexuality; it is not merely the absence of disease, dysfunction or infirmity. Sexual health requires a positive and respectful approach to sexuality and sexual relationships, as well as the possibility of having pleasurable and safe sexual experiences, free of coercion, discrimination and violence. For sexual health to be attained and maintained, the sexual rights of all persons must be respected, protected and fulfilled.^7^

In this expansive definition, the WHO indicates the inevitable connection between SH and sexuality: ‘a central aspect of being human throughout life, encompasses sex, gender identities and roles, sexual orientation, eroticism, pleasure, intimacy and reproduction’.^7^

The legitimacy of SH in the healthy ageing agenda is supported by evidence that sexual activity can continue into later life, albeit often at reduced frequency,^8–10^ and represents a correlate of quality of life.^10–13^ Quantitative work among European older adults and heterosexual couples reveals a positive relationship between ‘successful ageing’ and sexual satisfaction, validating sexual expression as a component of ageing well,^14,15^ consistent with scholarship among women.^16,17^ This growing body of evidence legitimizes SH as a tenet of ageing well, raising its prominence as a public health focus.^18^

Simon and Gagnon’s landmark 1986 publication introduced the theory that sexual behaviour is constructed according to sexual scripts based on pre-determined sociocultural norms, with variation in societal expectations according to gender, age and stage of the lifecycle.^19^ Women aged 50+ navigate sexual relationships under intersecting prejudices of ageism and sexism, with a gendered double standard in societal expectations of female physical appearance.^20^ Optimizing SH in a society that devalues older women presents challenges. Pervasive stigma and sociocultural norms may shape how women view their own SH and create barriers to health care engagement.^21^ Menopause and the end of fertility leaves women aged 50+ neglected in a health care agenda framed through a lens of reproductive function.^22,23^ Thus, gender-responsive SH research which interrogates the social landscape of ageing^23^ has been identified as a priority area for scholarship.^23,24^

Research investigating later-life SH typically applies quantitative methodology, condensing women’s sexual lives into reductive, dichotomous categories (sexually active/inactive, functional/dysfunctional)^25,26^ or applies a biomedical, disease-focused lens to examine impacts of ill health/disease/pathophysiology on sexual expression.^27^ There is a lack of qualitative research exploring female SH from an orientation of wellness,^27^ consistent with the WHO’s definition of SH.^7^ Nowakowski & Sumerau’s 2019 review identified a need for further examination of what SH means to women in later life.^24^ Furthermore, there has been a call for scholarship to move beyond discourses of decline to consider ‘pleasures of ageing’ and diversity in later-life sexuality.^28^

According to the 2021 census, 19% of the population of England and Wales were aged 65+, up from 16% in 2011.^29^ Supporting healthy ageing has been recognized as a priority within England’s National Health Service (NHS)^30^ and featured in the Chief Medical Officer for England’s 2023 report.^31^ An earlier (2015) report from the Chief Medical Officer specifically recognized SH as a component of the healthy ageing agenda and prompted by increased re-partnering and escalating sexually transmitted infection (STI) incidence among older adults.^32^ While quantitative scholarship in the United Kingdom (UK) has explored associations between sexual activity and well-being within nationally representative surveys,^11–13^ there is a lack of contemporary qualitative literature exploring SH in later life and its relevance to ageing well from an orientation of wellness.

The UN Decade of Healthy Ageing baseline report identified the need to strengthen the evidence base on determinants of healthy ageing to inform policy.^1^ This study sought to interrogate a ‘policy blind spot’ and examine interpretations and experiences of sexuality and SH within the context of ageing well among women aged 50+.^5,6^ This research builds on Gott and Hinchliff’s formative qualitative sociological scholarship exploring SH and ageing well in England in the 2000s,^33–35^ adding a feminist perspective in a modern landscape with increased international focus on healthy ageing.^1–3^

## Methodology

The qualitative study design was underpinned by an interpretivist epistemology, whereby knowledge derived from participants’ subjective accounts is considered legitimate.^36^ This study aligns with a naturalistic research paradigm by assuming multiple subjective realities, which are socially constructed. Under this paradigm, rich ‘real life’ context-bound findings emerge through mutual interaction between researcher and participant.^37^

### Theoretical frameworks

Research was guided by principles of feminist scholarship and located in an affirmative ageing framework. Feminist perspectives recognize women’s varied and complex experiences, perspectives and needs.^38–40^ This stands in contrast with an earlier tendency towards ‘malestream’ research, critiqued as overlooking or misrepresenting the experiences of women by applying an androcentric bias.^19,41,42^

An affirmative ageing framework challenges binary, reductive narratives of decline or success in ageing.^28^ Affirmative ageing theory views ageing as ‘the continuous production of difference’ and accepts the diversity in ageing journeys, acknowledging the gendered nature of ageing bodies and sexuality.^43^ This theory draws on the work of feminist corpomaterialists Grosz and Braidotti, who centralize the female body in theories of sexual difference.^44,45^

### Positionality

At the time of the study, the lead and second authors demonstrated strong research credentials (having both previously completed research-based doctoral degrees) and public health expertise (acquired through their occupations: Public Health Specialty Registrar and Lecturer in Public Health and Policy, respectively). Despite identifying as a woman, the lead author’s age at the time of data collection (mid-30s) located her as an ‘outsider’ among this older cohort. Younger researchers may encounter barriers when attempting to elicit frank discussions about later-life sexuality.^46^ Guided by previous research, interview questions were framed from a position of respect and desire to learn, to minimize negative ‘othering’ impacts.^46^

### Participants

Inclusion was limited to consenting English-speaking women aged 50+. Recognizing that women aged 50+ are heterogenous and identity is fluid across the life course, this research sought to capture the discursive nature of sexual expression in the context of ageing well by not limiting inclusion by health, relationship status or sexuality.

Decisions relating to the sample size were guided by achieving a depth of understanding related to the research question.^37^ Twenty-one participants were interviewed, at which point no new codes were being identified within the data, and it was determined that data saturation had been reached. This sample size reflects qualitative research norms^37^ and is in line with similar published work^25,35^ in which samples of this size have afforded sufficient data to be collected to meaningfully interpret the participants’ experiences. To reach a diverse group of women aged 50+ (unrelated to SH pathology), recruitment was mobilized from within three community organizations providing general information, advice and support to women in North West England. Prior to starting recruitment, the lead author established relationships with community organization managerial staff and clarified the purpose of the research and her credentials. Participation was extended to employees, volunteers and members using a combination of poster advertisements, in-person discussions and snowball sampling. Since recruitment, no participants have withdrawn from the study.

### Data collection

Consistent with a feminist interpretive epistemology,^47^ data were collected through semi-structured interviews, facilitating a moderately structured discussion, while retaining flexibility to explore and centre women’s diverse personal experiences.^37^ Interviews were constructed around an interview guide, which was informed by a literature review. The interview guide broadly explored: changes in sexual expression with ageing, perspectives on ageing well, the relationship between SH and ageing well and experience of SH provision in the context of ageing (see supplementary files for the interview guide, which had not been previously validated or pilot-tested). One-on-one interviews were conducted in private rooms at the community organizations between 18 April and 6 June 2019 and audio-recorded, lasting between 30 min to 1 h. No repeat interviews occurred. Reflexive notes were made by the lead author during the interview process.

### Data analysis

Data were transcribed using a denaturalized methodology, excluding ‘interview noise’ detracting from the main interview substance.^48^ Participants were assigned pseudonyms to preserve confidentiality. Data were analysed using a framework approach to thematic analysis, a method used to draw out patterns to explore connections and meaning within data, and generate interpretive insights and themes.^49^ A list of codes was produced through reading an initial portion of transcripts, with codes assigned to relevant phenomena. Codes were organized into larger categories to facilitate data management, and the coding framework was applied to all transcripts. A framework approach takes the thematic analysis further by tabulating summaries of relevant sections of data into matrices according to code and participant. This approach supports rigorous data interrogation, enabling the researcher to synthesize key points and identify patterns and gaps. Matrices were reviewed inductively to construct themes and subthemes derived from the data, minimizing the influence of the researcher’s positionality, personal research agenda or dominant sociocultural messaging.^50^ NVivo 12 software^51^ was used to facilitate the analytic process and manage the large data set.

The first author performed the interviews, transcription and analysis, promoting consistency across the research process. While arguably this increased the likelihood of bias, an interpretivist epistemology understands that the researcher’s analysis is an interpretation of participants’ interpretations (the double hermeneutic).^52^ To ensure the analysis reflected the participants’ spoken word, a reflexive approach was adopted throughout the analysis, with self-reflections documented and analysed within a reflective journal. Quotations were included to support interpretations.^53^ To enhance trustworthiness, the second author reviewed construction of the coding framework and its application to the transcripts.^54^ Discussions took place between the lead and second authors concerning the appropriateness of the coding strategy and the ability of the codes to capture phenomena in the data, with subsequent reviews of the matrices leading to refinement of final themes. Participants were not asked to review transcripts or provide feedback on findings.

## Results

Twenty-one interviews were conducted with women aged 52–76, generating 250+ pages of transcript. Participants were demographically diverse in income and employment status, but not in terms of sexuality and ethnicity, a limitation the authors reflect on in the discussion (Table 1).

**Table 1.** Participant characteristics (*n* = 21).

Characteristic	Number of participants (*n*)
Age	
50–59	10
60–69	9
70–79	2
Ethnicity	
White British	20
Asian British	1
Relationship status	
Married	8
Divorced, current partner	1
Divorced, no partner	9
Never married, no partner	3
Employment status	
Retired	10
Working	6
Unable to work due to ill health	5
Household annual income	
<£10,000	9
£10–20,000	5
£20–40,000	3
£40,000+	3
Menopausal status	
Peri-menopausal	2
Post-menopausal	19
Children	
Yes	16
No	5
Sexual orientation	
Heterosexual	21

Women’s narratives regarding SH and ageing well were captured within six broad themes (Table 2).

**Table 2.** Themes and subthemes derived from participant interviews.

Theme	Subtheme
Reflections on ‘ageing well’	Diverse accounts of ageing The relevance of sexual expression in ageing well
Age alone does not define sexuality and SH	Factors shaping later-life sexuality and SH Bidirectional impacts of ageing on sexuality and SH
Interpretations of SH and sexuality	Risk-based, disease-bound interpretations of SH and sexuality Recognizing SH as a tenet of general health and well-being
Vulnerability and resistance in later-life SH	Sexual risk in later life Sexual empowerment in later life
Narratives of (in)visibility	Societal surveillance of later-life SH and sexuality Invisibility of later-life SH in health care settings
Reimagining services to promote SH in later life	Barriers to discussing SH in health care settings Enabling holistic wellness through SH provision

### Reflections on ‘ageing well’

Accounts of ageing indicated varied experiences among this group of women. Almost all participants reported at least one mental or physical health concern; however, most remained socially and physically active and prioritized learning new skills. Some women associated ageing with pride and empowerment, expressing relief at shedding professional and childcare responsibilities, alongside societal expectations around body image that had previously constrained them. Age was perceived as irrelevant for some women. Penny, 67, remarked, ‘I don’t think of numbers. I don’t think, “oh you’re nearly 70”’. Others believed that understandings of what constitutes ‘older’ had evolved from previous generations, which they explained as being driven by increased healthy life expectancy.

Conversely, some women described a sense of lost value or status with advancing years, related to changing physical appearance, or lost physical or reproductive function. Others, such as Wendy, described challenges of growing older in a medicalized society, where ageing is pathologized and stigmatized.

I think we’re becoming an ageing population . . . at a time when it’s become more shameful, more difficult to be old . . . we’re made to feel ageing is a disease, and therefore every sign of ageing is just a symptom of the disease. (Wendy, 54)

Ageing well was conceived by women in terms of quality of life, and they described prioritizing healthy lifestyles, social connectedness and physical function. For some participants, sexual expression was viewed as an indicator of later-life physical function. Regardless of relationship status, most women believed sexual expression, whether partnered or alone, was an important part of ageing well. This contributed to affection within partnerships, mental and emotional well-being, general health and quality of life.

I think it’s really important because, I mean, that’s not to say somebody who is not having a sexual relationship does not age well [. . .] I think if I was in this relationship and it was withdrawn, the sexual part of it, I would start feeling less desirable. And that would make me feel less of a woman and unstable, and I’d also miss it. I’d miss the intimacy and I’d miss the sex and I’d miss the pleasure that gives you, you know, the physical pleasure as well as the emotional, you know, enrichment etcetera. But I think I would start to feel less of a woman. (Wendy, 54)

In this thinking, sexual expression is conceived of as an enriching aspect, rather than a requirement, of ageing well. Many women rated companionship and emotional connection above physical sexual activities in contributing to well-being. However, some perceived sexual expression as less, or not at all, important when compared to other concerns. Furthermore, some described experiencing negative impacts of sexual relationships, including distress driven by incompatible partnerships, or painful intercourse: ‘It [intercourse] was always hurting me . . . I don’t know why’ (Beth, 60). For some, the ending of an unhappy or abusive relationship was a catalyst for improved quality of life.

### Age alone does not define sexuality and SH

Interpretations of sexuality were wide ranging, encompassing sexual orientation, appearance, femininity, desire and need. However, women were largely united in their understanding of sexuality as a natural and intrinsic part of their identity, as expressed by Olivia, 62:

I suppose it’s like a person’s – not soul – [. . .] a little bit of what’s inside them, and it can be linked to positivity, can’t it? And I suppose . . . not thinking that you’re old and past it . . . it’s not something I feel shame about or anything like that, we’re all getting those desires [. . .] It’s part of who you are.

Sexual expression was understood as fluid across the life course, varying in response to health problems, life circumstances, caring responsibilities, menopause, relationship factors and, to some degree, age. Less than half of women regularly engaged in partnered sexual activities, which was largely, but not entirely, described as being driven by relationship status. Forms of sexual expression varied in response to physical or health limitations or personal preferences, with some deprioritizing penetrative intercourse in favour of alternative forms of partnered intimacy, and others enjoying solo acts of sexual expression.

Now and again I find that if you don’t have anything to do with sex, you do feel a bit dull, so I think it’s good to explore yourself, a woman on her own to explore herself. (Alice, 68)

For some women, relationship difficulties or health problems meant partnerships lacked sexual intimacy. Some women preferred not to engage in sexual relationships, while others, including May, described being denied sexual intimacy within partnerships.

I’ve been married since 1996 [. . .] for me, we only had intercourse that year, and we haven’t had any since, after that, because, because my husband was impotent [. . .] I used to get quite angry, really, and frustrated. But now I’ve sort of accepted it, really. But I think it is quite important, really [. . .] I used to get really angry, but I accept it now . . . You know if, like, I could’ve spoken to somebody, it might’ve helped. (May, 57)

For some women, the menopause transition triggered psychological symptoms or physical changes, representing a challenging time for their sexuality, womanhood and personal identity. For others, it exacted minimal symptoms and had no impact on their sexual lives. However, many women reported liberation post-menopausally and freedom from concerns about contraception and menstruation.

Age was understood as only one of a great many factors shaping later-life sexuality. For some women, advancing age empowered them with the confidence to enjoy new sexual freedoms, or to resist pressures to conform to sexual norms, as expressed by Nancy, 59:

I’m really just not interested in having [. . .] sexual relationships. I find it all a bit of a bore, to be honest. And I have for a while. But I wouldn’t admit it to myself. And then there came a space where, God, this is . . . it’s freedom, it’s like I don’t have to bother with all this nonsense anymore. It was fun while it lasted . . . and if that’s part of getting old, bully for it!

Conversely, ageing was described as having negatively impacted sexual expression for others, driven by altered physical form. However, a concern emerged that changes in sexual expression due to poor health or menopause may be incorrectly attributed to age.

Well, if people have got problems [. . .] they think, oh it’s all part and parcel . . . it’s just my age. (Helen, 76)

In Helen’s telling, a willingness to attribute SH problems to age may hinder help-seeking. At the same time, while women in this study felt that ageing had not changed their basic SH needs, they noted SH assuming a lower priority in their interaction with health care professionals once they had transitioned out of the reproductive years.

### Interpretations of SH and sexuality

Most women defined SH in risk-based terms, related to STIs and cervical screening. For some, the origin of narrow conceptualizations of SH could be located in earlier years, with discussions about sex described as absent when younger, or veiled in embarrassment and shame. An ambivalent relationship with SH clinics also emerged. While some women expressed a willingness to access SH clinics, others felt deterred, with many believing the service was targeted towards the young, or stigmatized due to its association with STIs: ‘When you say sexual health clinic, you tend to think of things that you need treatment for . . . It does put you off’ (May, 57).

Others constructed a broader picture of SH, incorporating elements of sexual desire, satisfaction and agency, body image, menopause and general well-being.

I know primarily we think about protecting ourselves against certain sexually contracted diseases [. . .] But I suppose, actually, it’s about being confident and comfortable to make sure [that] it is fully consensual . . . that’s healthy sex. Because if it’s not that, then you’re doing stuff against your will [. . .] Or you’re doing something just to please somebody else [. . .] You’re not taking care of your own health, and your sexual health, just like your mental health and physical health. It’s all part of the same thing [. . .] If you do something badly in one area, it’s going to impact on another. (Wendy, 54)

Despite many women recognizing SH as a tenet of general well-being in this way, SH was perceived as being separate from, and even prioritized below, general health in medical settings. Participants described SH being approached from a disease-focused lens during consultations. Others believed that SH was over-medicalized, in both clinical and social settings.

If you turn around to somebody and say, I’m really not that interested in sex, oh, there’s something terribly wrong with you! There’s something affecting your libido, you must be terribly depressed! Yeah, it could be a warning sign of that, just like 1001 things are; but why do people automatically jump [to that conclusion]? (Nancy, 59)

In the main, when asked about ‘SH needs’ during the interview, women understood this as meaning engagement with cervical screening programmes. For some, this held negative connotations due to unpleasant experiences of cervical screening.

### Vulnerability and resistance in later-life SH

Women’s narratives conveyed competing accounts of vulnerability and resistance in later-life SH. Stories of abusive or controlling relationships and sexual violence featured in many women’s lives. Those affected demonstrated resilience, whether or not they chose to pursue other relationships. Not all experiences were historical, with one woman describing a recent incident where she was sexually assaulted in her 50s while discovering her identity as a newly single woman.

Some women described empowerment in later life, exhibiting greater sexual agency in selecting partners and prioritizing safer sex. For others, transitioning out of the reproductive model of SH changed their perceptions of sexual risk, SH and condom negotiation:

There’s something sexy and interesting about not getting pregnant . . . [But] because I don’t use the pill, I use condoms, it was like – Oh, I can’t say I want to use a condom because I don’t want to get pregnant, so I’ve now got to say ‘in case you’ve got an STI’, which is not sexy . . . It changes the dialogue. (Sharon, 52)

In some cases, narratives of invulnerability stemmed from inadequate foundations in SH knowledge earlier on in the life course. As described above, SH education was often absent or framed from a reproductive lens:

Nobody ever used to talk about diseases and things like that, it would be more the reproductive system and how babies were made. Yeah, you never heard about like, chlamydia and all these things that are going round now. And that was in secondary school, we never got taught nothing like that. Condoms! You wouldn’t even know what a condom was . . . I came to a course in here [the community organisation] 13 years ago, and never knew there was so much contraception. I couldn’t believe it, and I had two grown up kids . . . I didn’t even know what the GUM clinic was! (Rachael, 54)

Some women associated maturity with increased sexual vulnerability (both physically and emotionally), and many expressed concerns about a growing laissez-faire attitude to sexual risk among older generations.

You’ve got women my age and older that think they are invincible, and they’re taking horrendous chances that if their daughters or granddaughters were doing the same, they would be horrified . . . I think it’s going to be an explosion . . . there’s a lot of people trying to relive their youth, in old bodies [laughs] (Tracey, 55)

Despite demonstrating an awareness of STI risks, some women revealed they did not use condoms. This choice was explained by an assumed invulnerability (acquired through years of successfully avoiding STIs), or the belief that age itself conferred a degree of protection from sexual risk.

Women’s accounts suggested that increased social and technological mobility represented a vehicle for enhanced sexual liberation on one hand, and increased sexual risk in later life on the other. Single women described engaging in sexual partnerships abroad, through holidays or singles cruises. Many married women perceived online dating to be dangerous and a threat to the SH of older single women. Those engaging in online dating recounted mixed experiences. Some found it easier to exercise sexual agency and establish new relationships through online platforms. As noted by Debbie, 62, ‘Where does a woman go at that age to meet a person? Men can go to a pub on their own and sit there. Women . . . it’s hard’. For others, online dating was perceived to increase disposable partnerships, present another conduit for sexual manipulation by men and increase scrutiny of physical appearance and age within dating profiles. Others believed it shifted the boundaries of appropriate behaviour.

There’s a lot of younger guys who want an older woman, and who want a woman who’s dominating. I’ve never done that, so I don’t know. But the number of guys – God, if I had a pound for everyone who’s said . . . I’m looking for someone to dominate me, small punishments [or] withholding orgasm from them. (Sharon, 52)

As highlighted by Sharon, the tension between sexual empowerment and vulnerability to sexual risks in later life was augmented by the expansion of digital dating platforms.

### Narratives of (in)visibility

Opposing narratives emerged regarding societal surveillance of later-life SH, shaping women’s sexual lives in different ways. Some women suggested that later-life sexual expression was overshadowed by gendered double standards in societal expectations of female body image and later-life (a)sexuality.

We know that we have to live in the society we live in, so even if you go ‘I don’t give a damn what you think, I’m going to age disgracefully, do whatever I want to do, be totally sexual, sexualised’, we know we live in a society that, you know, [is] laughing, pointing, staring, dismissing, devaluing, finding it inappropriate. And it’s really hard to be so thick skinned that you don’t care about that. (Wendy, 54)

While others were unaware of, or unaffected by, societal constructions or assumptions about later-life sexuality, some described a hyper-visibility of sexuality, with society’s preoccupation painted as obsessive, unhealthy and controlling. While there was a general belief that awareness, acceptability and openness around SH and sexuality were improving, this was not consistently felt to have extended to older adults. Other women suggested sexual liberation had not yet been ‘truly realised’ by women of any age.

We have so many more freedoms than we did, and those freedoms, rather than us genuinely enjoying them, have been exploited to make us paranoid and miserable. It’s become another thing to beat ourselves with as opposed to celebrate . . . I think their [women’s] attitude is almost like my history teacher said, revolution, reaction, then reform. We’ve had the revolution, we are in the middle of the reaction, and then eventually, maybe in 50–100 years’ time, we will have that genuine [sexual] liberation where we take it for granted, we don’t have to obsess about it, and we don’t have to shove it under the carpet. (Nancy, 59)

Most women felt evolving digital landscapes had reimagined the boundaries of later-life sexual surveillance. For example, many women perceived later-life SH now receiving more visibility in the media. While some perceived this as an advantage through increased awareness, others believed it perpetuated unrealistic expectations about sexuality and body image. Some women commented that navigating sexual relationships through online dating augmented the surveillance ‘atmosphere’, with female physical appearance and age subject to extra scrutiny from potential partners through their online profile. As expressed by Isobel, 62: ‘I got asked the question yesterday by someone I’m only talking to [on an online dating site], “Do you still have sex?” I was like, “just because I’m 62, doesn’t mean to say I can’t”’.

In contrast, as described above, women perceived SH as becoming less visible in health care settings as they aged out of the reproductive model of SH. The invisibility of SH surveillance in the health care agenda for older women was accentuated by the reduced frequency of cervical screening beyond age 50 in England, eventually stopping at 64. For many women, this represented the only form of SH follow-up.

The older you get, the less likely you are to be on the radar of a lot of these [. . .] things. It’s, ‘Ooh, what do you want to be bothered about that for’? I mean, I’m fit and healthy, so why would a doctor want me to go to him and say, well actually [. . .] unless I had a problem, you know [. . .] Obviously, you go for your [cervical] smear test and all the rest of it, and then they don’t . . . and even that, you come to an age and they say ‘right, you don’t have them anymore’ and I don’t think that’s right. (Debbie, 66)

While some women had approached their general practitioner (GP) with SH concerns during menopause, GPs had not proactively asked any of the participants about SH in later life. Women reported that discussions of this nature were similarly absent from NHS Health Checks. Most participants considered that time pressures and competing health priorities may drive the reduced visibility of SH in primary care settings. However, others believed ageist assumptions around later-life sexual expression also played a role. Indeed, some women reported experiencing explicit ageism within health care settings. Emily, 61, recounted:

So, I went [to the sexual health clinic] after work one night [. . .] and it was all these kids in there. Youngsters. And when I got up this lad turned round and – I’ll always remember – he said to me, ‘You’re a bit old for in here, aren’t you?’

Ageist labels and social constructions of older women as unsuitable recipients of SH care posed additional barriers to engaging with services for some.

### Reimagining services to promote SH in later life

While most women described feeling comfortable approaching their GP with problems, there was a lack of clarity around what SH provision was available to women in later life. Women experienced various barriers to discussing SH in primary care settings, including embarrassment, not perceiving it as a ‘medical’ concern, time pressure, male doctors and uncertainty related to the health care provider they would encounter. The majority of women believed more could be done to actively promote SH in later life. As expressed by Valerie, 59: ‘It [SH provision] needs to be more proactive because at the end of the day, women either suffer in silence or can’t talk about it’.

In reimagining service improvement, a dominant preference for women-led, nurse-led, age-specific clinics emerged as a means to promote accessible, appropriate and holistic health care delivery: ‘Like a well women clinic, or something like that’ (Debbie, 66). Many women felt that attendance would be more discreet if clinics were housed within a GP surgery, rather than a specific SH facility. A strong preference emerged that later-life SH provision should be recognized as a component of holistic wellness, and incorporated under the umbrella of routine health checks in later life.

It’s a bit like incorporating the post office at your local library isn’t it? That seems like the obvious time to do it [. . .] if you’re doing a general health check up, which you do at certain ages don’t you. That should be part of it, shouldn’t it? Why is it not? [. . .] It reinforces the fact that you shouldn’t be, it’s not a problem for you. ‘You don’t need to worry about it’. (Wendy, 54)

Most women expressed a desire to mobilize information sharing within non-clinical settings to disrupt narrow, disease-focused interpretations of SH and normalize sexuality in later life. Informal group consultations or speaker sessions were suggested to aid holistic discussions that were unbound to sexual problems, prioritizing conversations around ‘normal’ ageing, sexuality and menopause within a non-medicalized space.

## Discussion

In the context of an ageing global population with an increased international spotlight on ‘healthy ageing’,^1–3^ this study fills knowledge gaps related to the understanding of women’s SH in later life and its relevance for ageing well.^24^ Exploring female SH from an orientation of wellness is a critical corrective to the problem-based focus of much literature on later-life sex.

Sexual expression was regarded by many participants as an important component of ageing well, regardless of the range or frequency of sexual experiences. Sexual expression was described as promoting emotional intimacy, psychological well-being and quality of life, or indicating continued physical function. Despite its perceived contribution to ageing well, many participants reported positive well-being and quality of life in the absence of sexual expression. Equally, the *absence* of a damaging sexual relationship facilitated ageing well for some. This raises a significant caveat, that while sexual expression may enhance experiences of ageing well, it is not a requirement, and may even compromise ageing well for others. Any movement to incorporate SH into the healthy ageing agenda must avoid perpetuating a marginalization of women who do not conform to prescriptive sexual hierarchies or acceptable femininities.^55^

Ezhova’s scoping review illuminated ongoing stigmatization of later-life sexual expression.^56^ Similarly, in our study conflicting social norms overshadowed later-life sexual expression, with stigmatizing asexual stereotypes competing against ‘Positive Ageing’ narratives, whereby women aged 50+ are bound by youth-orientated sexual and physical standards.^57,58^ The Positive Ageing Movement rebelled against traditional views of age-related decline and challenged the dominant (a)sexual script assigned to older women, reimagining later-life sexuality as unaffected by ageing (or ameliorated with treatment).^57,58^ However, this movement has been criticized for being tethered to biomedical foundations, portraying ‘successful ageing’ and ill health as mutually exclusive and placing unrealistic sexual expectations on older adults, marginalizing those who fail to age well by exhibiting ‘dysfunctional’ sexual performance.^59,60^ These competing ideals advance hegemonic models of sexual functional, while simultaneously pathologizing ageing bodies.

Women’s recognition of SH as part of ageing well was illustrated by their preference to incorporate SH discussions into routine health checks. A desire for person-centred holistic provision emerged, which f﻿ocusses on advancing well-being rather than addressing dysfunction. This perspective is consistent with Tiefer’s ‘new view’ paradigm of female sexuality, which supports a rights-based, life course approach to SH, unbound from sexual pathology and reflecting the nuanced complexity of lived experience.^61^ Incorporating SH discussions as part of integrated women’s health provision^62^ could normalize and legitimize these discussions, destabilizing the deficit model of women’s later-life SH characterized in many participants’ accounts. This approach would help to support a move towards ‘person-centred, integrated care and primary health services responsive to older people’, a priority action of the UN Decade of Healthy Ageing.^2^

The menopause consultation may be an opportune time to assess women’s SH needs and initiate sex-positive post-menopausal care.^63^ However, participants recalled SH discussions as being largely absent in health care encounters during the menopause transition. Feminist scholars argue that SH is narrowly conceptualized through a reproductive lens, disregarding any motivation for sexual expression beyond procreation.^23^ This perpetuates ageist myths that sexuality expires post-menopause and propagates a homogenization of women in later life. These findings support calls to adopt an equitable life course approach to optimize SH and rights for all women.^23^

In women’s narratives, the Internet emerged as both an enabler and a threat to later-life SH. With digital literacy increasing among older adults in the UK,^64^ the Internet has risen in prominence as a discreet platform for establishing romantic connections amid shifting perceptions of acceptable social environments for older women. While research contends that digital spaces can increase sexual agency and empowerment,^65^ critics argue this shift represents (yet another) mechanism through which older women are sexually devalued, perpetuating ageist stereotypes, and propagating sexual risk.^66,67^ This study supports calls for SH provision to adapt to the needs of women navigating sexual partnerships in later life in an increasingly ‘digitised’ world.^60^

Notably, sexual trauma permeated through the lives of many participants. The prevalence and impacts of sexual violence have traditionally been overlooked or underestimated within the literature on later-life SH.^68,69^ However, an analysis of police reports in England, Wales and Northern Ireland highlights the magnitude and consequences of sexual violence among older women.^69^ Given that sexual trauma has been linked to adverse SH outcomes in later life,^70^ sensitively advancing trauma-aware SH provision for older women is a priority. Intersectional research interrogating experiences and consequences of sexual trauma in later life is indicated.

Our findings advocate for enabling environments to legitimize the diversity of later-life sexual expression and enhance women’s ageing journeys. Empowerment through education and awareness is critical, and dissemination of SH information should extend beyond clinical settings to advance sexual agency and resilience.^71,72^ Finally, this study draws attention to a broader need to mobilize social change to disrupt societally defined hierarchical structures that propagate ageist inequalities. Our findings lend support to priority areas of action identified within the UN Decade of Healthy Ageing vision to optimize the experience of ageing, including challenging ageist attitudes and supporting later-life autonomy, dignity and well-being.^2^

### Strengths and limitations

As this research specifically examined interpretations and experiences of sexuality and SH within the context of ageing well in England, findings may not be generalizable to settings with different sociocultural influences, policies and health care infrastructure. However, given global commitments to support healthy ageing,^1–3^ this study raises important conclusions related to the relationship between SH and ageing well among women, which are relevant on an international scale. Qualitative methodology increased the depth and flexibility of the analysis, enriching women’s diverse sexual narratives with a life course perspective.^37^

All participants attended community organizations; thus, perspectives of women not engaged with community organizations are not captured, as well as those unable to attend owing to competing priorities, health problems or disability.

The sample was not sufficiently diverse to fully interrogate different axes of identity that may shape women’s experiences of SH and ageing. As no participants were aged 77+, the perspectives of the oldest women are absent. While this may be driven by an historical reluctance of older generations to discuss SH, immobility or ill health may have also limited community organization attendance. The absence of participants identifying as sexual and gender minorities means the lived experience of women whose sexual orientation and behaviours may be subject to an additional layer of societal stigma is lacking. Similarly, the voices of ethnic minority women are underrepresented. An intersectional analysis^73^ interrogating how axes of identity and social position shape women’s experiences of SH is indicated to strengthen this knowledge foundation and further define optimal SH provision for the diverse cohort of women ageing in England.

## Conclusion

This research represents an important contribution to the literature on women’s SH and sexuality in later life and supports their relevance for the global healthy ageing agenda.^1–3^ Among in-depth interviews with 21 women aged 50+, SH represented a contributor to ageing well for many, despite a broad spectrum of sexual expression. Sexual expression was diversely shaped by health challenges, menopause, life experiences and partner factors, overshadowed by societal expectations within a shifting digitized environment. Damaging relationships, sexual trauma and suboptimal SH education across the life course-reinforced calls to build resilience, agency and empowerment through holistic SH care; however, these discussions were perceived as muted or problematized in clinical settings. A preference emerged to reimagine person-centred SH provision, uncoupled from disease/dysfunction and sensitive to diverse sexual, health, life and relationship realities. This article strengthens calls to legitimize SH as part of the healthy ageing agenda.

## Supplemental Material

sj-docx-1-whe-10.1177_17455057241247747 – Supplemental material for Love (and) ageing well: A qualitative study of sexual health in the context of ageing well among women aged 50 and over Supplemental material, sj-docx-1-whe-10.1177_17455057241247747 for Love (and) ageing well: A qualitative study of sexual health in the context of ageing well among women aged 50 and over by Sophie Patterson and Kate Jehan in Women’s Health

## Declarations

**Ethics approval and consent to participate:** Ethical approval was granted from the Health and Life Sciences Research Ethics Committee at Liverpool University in July 2018 (reference: 3682). All participants were provided with an information sheet detailing the title, purpose and motivation of the study (i.e. in pursuit of a Master of Public Health), the voluntary nature of participation (with option to withdraw at any time), what being a participant would entail (including risks and benefits of participating), contact information of research team and research ethics and integrity office, signposting to additional support, confidentiality protocols, and how results will be used. After reviewing the participant information sheet and providing the opportunity to ask questions, written informed consent was obtained in person by the interviewer.

**Consent for publication:** Participants were informed in the information sheet that anonymized research findings may be published, and written consent was given based on this understanding.

**Availability of data and materials:** Data requests should be made through the corresponding author.
