# Supplementary material for: Love (and) ageing well: A qualitative study of sexual health in the context of ageing well among women aged 50 and over
Source: Womens Health (Lond). 2024 Apr 29;20:17455057241247747. doi: 10.1177/17455057241247747 (PMC11057352; doi:10.1177/17455057241247747)
Supplement: sj-docx-1-whe-10.1177_17455057241247747 – Supplemental material for Love (and) ageing well: A qualitative study of sexual health in the context of ageing well among women aged 50 and over [file sj-docx-1-whe-10.1177_17455057241247747.docx]

**
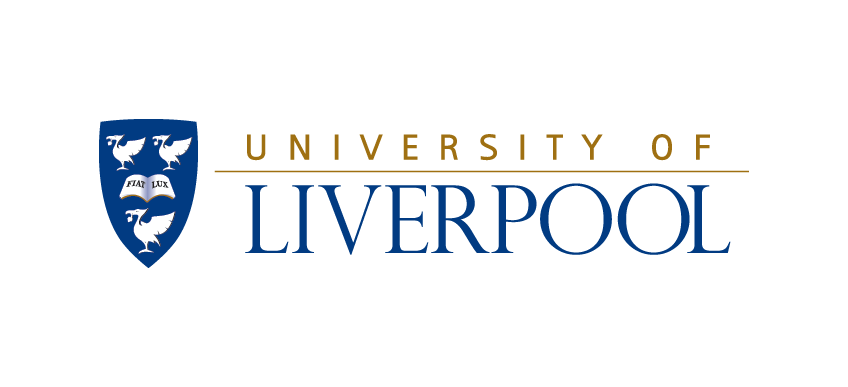
**


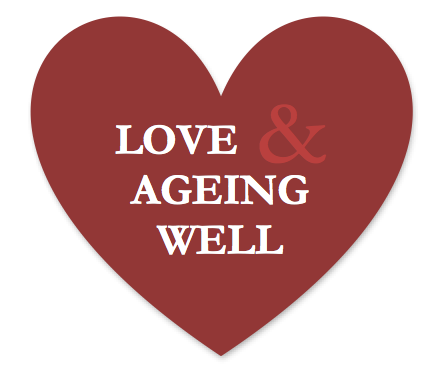


**Interview Topic Guide**

**1. To start with, I’d like to learn more about you. Please could you tell me a bit about yourself?**

- Can you tell me about your home and family circumstances*? Prompts: where do you live? Do you live alone? Do you have children/grandchildren?*
- Can you tell me about how you would spend a typical day? *Prompts: Retired? Volunteering? Responsible for caring for a partner or relative with poor health? Social connectedness?*
- Can you tell me a little about your health? *Prompts: Do you have any physical or mental health problems that limit your daily activities? Do you take regular medications? Have you been through the menopause?* [If yes] *what was your experience of the menopause? Timing of menopause?*

**2. Now I’d like you to talk to me about your relationships.**

- [If currently in a romantic partnership] can you tell me about your current relationship? *Prompts*: Is this a regular partnership? *Duration of relationship?* *What sex is your partner? What forms of intimacy do you and your partner engage in? Are you satisfied in your current relationship?* *Preferred contraceptive methods?*
- Tell me a little about your previous relationships? *Prompts: sex of previous partners? Where they regular partner(s)? What forms of intimacy did you and your partner(s) engage in?*

**3. Could you tell me a little about how your sexual life has changed as you have become older?**

- As you have become older, what aspects of your sexual life have improved, if any? *Prompts: considerations about contraceptive choice?* *Considerations about pregnancy? Enjoyment and satisfaction?*
- As you have become older, what challenges have you faced in maintaining a healthy and satisfying sexual life, if any? *Prompts: Considerations around libido, discomfort? Partner related challenges?*
- [If postmenopausal] Can you tell me what impact, if any, the menopause has had on your sexual life?

**4.** **This research explores sexual health in the context of ageing. From your perspective, do you believe sexual wellbeing is an important component of healthy ageing for women? If so/not, why?**

- Do you think other women in your community would agree with your view? Please elaborate.
- Do you think healthcare providers would agree with your view? Please elaborate.

**5.** **Now we will move on to explore your experience of sexual health care provision**

- Could you talk to me a little about how your sexual health care needs have changed as you have become older? *Prompts: Do you feel your sexual health care needs as an older woman are currently met? What aspects of your sexual life, if any, do you feel would benefit from healthcare intervention?*
- Could you talk to me a little about how your sexual health care provision has changed as you have become older? *Prompts: Can you tell me a little about your experiences of sexual health discussions during consultations with healthcare providers as an older woman*? *Are sexual health discussions more or less commonplace in healthcare settings compared to earlier in the life course? When did your GP last ask you about your sexual health as part of your routine care?*
- Can you tell me a little more about which healthcare providers you would feel comfortable talking to about issues of sexual health and ageing as an older woman? *Prompts: GP? Gynaecologist? Sexual health clinic? Which healthcare provider would you prefer to go to if you had a concern about your sexual health?*
- Could you talk a bit about your expectations of how sexual health care provision for older women could be improved? *Prompts: in GP setting? In hospital settings? In community settings?*

**6. I’ve really enjoyed listening to your views and experiences – is there anything else you would like to add that you feel is important on the topic and we have not covered today?**

***NB: This is a dynamic document and is subject to change during the research process.***
